# Monthly Sulfadoxine-Pyrimethamine During Pregnancy Prevents Febrile Respiratory Illnesses: A Secondary Analysis of a Malaria Chemoprevention Trial in Uganda

**DOI:** 10.1093/ofid/ofae143

**Published:** 2024-03-13

**Authors:** Jordan John Lee, Abel Kakuru, Karen B Jacobson, Moses R Kamya, Richard Kajubi, Anju Ranjit, Stephanie L Gaw, Julie Parsonnet, Jade Benjamin-Chung, Grant Dorsey, Prasanna Jagannathan, Michelle E Roh

**Affiliations:** Division of Infectious Diseases and Geographic Medicine, Department of Medicine, Stanford University, Stanford, California, USA; Department of Epidemiology and Population Health, Stanford University, Stanford, California, USA; Infectious Diseases Research Collaboration, Kampala, Uganda; Division of Infectious Diseases and Geographic Medicine, Department of Medicine, Stanford University, Stanford, California, USA; Kaiser Permanente Northern California Division of Research, Vaccine Study Center, Oakland, California, USA; Infectious Diseases Research Collaboration, Kampala, Uganda; Department of Medicine, Makerere University, Kampala, Uganda; Infectious Diseases Research Collaboration, Kampala, Uganda; Division of Maternal-Fetal Medicine, Department of Obstetrics, Gynecology, and Reproductive Sciences, University of California San Francisco, San Francisco, California, USA; Division of Maternal-Fetal Medicine, Department of Obstetrics, Gynecology, and Reproductive Sciences, University of California San Francisco, San Francisco, California, USA; Division of Infectious Diseases and Geographic Medicine, Department of Medicine, Stanford University, Stanford, California, USA; Department of Epidemiology and Population Health, Stanford University, Stanford, California, USA; Department of Epidemiology and Population Health, Stanford University, Stanford, California, USA; Chan Zuckerberg Biohub, San Francisco, California, USA; Division of HIV, Infectious Diseases, and Global Medicine, Department of Medicine, University of California San Francisco, San Francisco, California, USA; Division of Infectious Diseases and Geographic Medicine, Department of Medicine, Stanford University, Stanford, California, USA; Department of Microbiology and Immunology, Stanford University, Stanford, California, USA; Department of Epidemiology and Biostatistics, University of California San Francisco, San Francisco, California, USA; Malaria Elimination Initiative, Institute for Global Health Sciences, University of California San Francisco, San Francisco, California, USA

**Keywords:** antibiotics, dihydroartemisinin-piperaquine, intermittent preventive treatment of malaria in pregnancy, nonmalarial fevers, sulfadoxine-pyrimethamine

## Abstract

**Background:**

Trials evaluating antimalarials for intermittent preventive treatment in pregnancy (IPTp) have shown that dihydroartemisinin-piperaquine (DP) is a more efficacious antimalarial than sulfadoxine-pyrimethamine (SP); however, SP is associated with higher birthweight, suggesting that SP demonstrates “nonmalarial” effects. Chemoprevention of nonmalarial febrile illnesses (NMFIs) was explored as a possible mechanism.

**Methods:**

In this secondary analysis, we leveraged data from 654 pregnant Ugandan women without HIV infection who participated in a randomized controlled trial comparing monthly IPTp-SP with IPTp-DP. Women were enrolled between 12 and 20 gestational weeks and followed through delivery. NMFIs were measured by active and passive surveillance and defined by the absence of malaria parasitemia. We quantified associations among IPTp regimens, incident NMFIs, antibiotic prescriptions, and birthweight.

**Results:**

Mean “birthweight for gestational age” *Z* scores were 0.189 points (95% CI, .045–.333) higher in women randomized to IPTp-SP vs IPTp-DP. Women randomized to IPTp-SP had fewer incident NMFIs (incidence rate ratio, 0.74; 95% CI, .58–.95), mainly respiratory NMFIs (incidence rate ratio, 0.69; 95% CI, .48–1.00), vs IPTp-DP. Counterintuitively, respiratory NMFI incidence was positively correlated with birthweight in multigravidae. In total 75% of respiratory NMFIs were treated with antibiotics. Although overall antibiotic prescriptions were similar between arms, for each antibiotic prescribed, “birthweight for gestational age” *Z* scores increased by 0.038 points (95% CI, .001–.074).

**Conclusions:**

Monthly IPTp-SP was associated with reduced respiratory NMFI incidence, revealing a potential nonmalarial mechanism of SP and supporting current World Health Organization recommendations for IPTp-SP, even in areas with high-grade SP resistance. While maternal respiratory NMFIs are known risk factors of lower birthweight, most women in our study were presumptively treated with antibiotics, masking the potential benefit of SP on birthweight mediated through preventing respiratory NMFIs.

## Teaser

Intermittent preventive treatment in pregnancy with sulfadoxine-pyrimethamine is associated with improved birthweight outcomes despite limited antimalarial activity. In this secondary analysis, we report that sulfadoxine-pyrimethamine is associated with reduced risk of respiratory illnesses during pregnancy.

Malaria in pregnancy is a major cause of adverse birth outcomes, including low birthweight and preterm birth in sub-Saharan Africa [1, 2]. For pregnant women living in malaria-endemic countries, the World Health Organization recommends intermittent preventive treatment in pregnancy (IPTp) with sulfadoxine-pyrimethamine (SP) starting from the second trimester of pregnancy to delivery [2]. This recommendation, established in 2004, was based on several randomized controlled trials demonstrating the efficacy of IPTp with SP on reducing maternal anemia, placental parasitemia, and low birthweight [3, 4]. However, over the past 2 decades, the rise of *Plasmodium falciparum* resistance to SP in eastern and southern Africa [5, 6] has prompted research into identifying alternative drugs for IPTp. An attractive alternative to SP is dihydroartemisinin-piperaquine (DP), an artemisinin-based combination therapy shown to be safe and highly effective against malaria in pregnancy [7, 8]. Five clinical trials conducted in areas of high SP resistance found marked reductions in the incidence of malaria among pregnant women receiving IPTp with DP as compared with IPTp with SP [9–13]. Yet, in many of these studies, this greater decrease in malaria incidence did not translate into better birth outcomes such that women receiving IPTp with DP had lower birthweight babies when compared with women receiving IPTp with SP [9, 13, 14].

One likely reason for this contradictory finding is that IPTp-SP may exhibit nonmalarial benefits on birth outcomes. In addition to its antimalarial activity, SP has demonstrated antibacterial activity against *Staphylococcus aureus*, *Streptococcus pneumoniae*, *Escherichia coli*, and *Enterobacter* species [15–17], sharing similar characteristics with trimethoprim-sulfamethoxazole, an antibiotic used to treat many bacterial infections, including acute respiratory illnesses, urinary tract infections, diarrhea, and other fever-inducing bacterial infections [18, 19]. More recent evidence has found that when compared with IPTp-DP, IPTp-SP may play an important role in preventing sexually transmitted infections and reproductive tract infections [20] such as *Chlamydia trachomatis* [9], reducing risk of enteroaggregative *E coli* [15], preventing enteric dysfunction and intestinal inflammation [21], and facilitating higher maternal weight gain [9, 15]. Indeed, SP's broad antimicrobial activity may have impacts on numerous nonmalarial infections and/or conditions, which in turn may affect more downstream outcomes, such as birthweight.

In malaria-endemic settings where IPTp is recommended, fevers are relatively common and generally attributed to malaria. Fevers caused by other pathogens or nonmalarial fevers are also common in these settings yet difficult to specify due to diagnostic limitations [22]. While the causes of febrile episodes may be multifactorial, the occurrence of fever during pregnancy has been associated with poor birth outcomes and long-term physical and neurodevelopmental disorders in the infant, though findings have been mixed [23, 24]. Because of concerns for co-occurring illnesses [22, 25], antibiotics are often prescribed presumptively with antimalarials for febrile illnesses in malaria-endemic settings, which may also affect birth outcomes [26, 27]. The impact of IPTp regimens on nonmalarial fevers and antibiotics and whether these factors may mediate effects of IPTp regimens on differential birth outcomes remains unclear.

Here, we examine whether IPTp-SP is associated with reducing nonmalarial febrile illnesses (NMFIs) and whether SP or other antibiotics prescribed during pregnancy improve birthweight. To understand these relationships, we leveraged data from a clinical trial conducted in Busia, Uganda (NCT02793622), and used novel causal mediation analyses to examine the extent to which the incidence of NMFIs and treatment via antibiotics mediate the greater impact of IPTp-SP on birthweight outcomes when compared with IPTp-DP.

## METHODS

### Study Population

We utilized individual-level data from a double-blind randomized placebo-controlled trial comparing the efficacy of DP vs SP for IPTp. The study took place in Busia District, a rural area of eastern Uganda with perennial malaria transmission. Pregnant women who were at least 16 years of age and between 12 and 20 weeks of gestation were eligible for enrollment. Exclusion criteria were HIV infection, receipt of prior IPTp in their current pregnancy, and residence outside of Busia District at the time of enrollment.

Detailed trial procedures are described elsewhere [11]. Briefly, women were randomized to receive monthly IPTp with either SP or DP starting at 16 or 20 weeks of gestation, depending on gestational age at enrollment, and to continue it through delivery. Each course of SP comprised 3 tablets of 500-mg sulfadoxine and 25-mg pyrimethamine. Each course of DP comprised 3 tablets of 40-mg dihydroartemisinin and 320-mg piperaquine given once a day for 3 consecutive days. Placebos were used such that participants received either active SP plus placebo DP or placebo SP plus active DP. The single course of SP and the first dose of DP were directly observed, while the second and third doses of DP or placebos were taken at home. Adherence to study drugs self-administered at home was determined by participant recall at the subsequent monthly clinic visit.

For our analyses, only women with singleton live births who received ≥1 IPTp courses were included. Women contributed study time from the date of their first IPTp course until delivery. Women were excluded if they withdrew from the trial before delivery or experienced fetal loss.

Starting from the date of their first IPTp course until delivery, participants engaged in routine visits every 4 weeks that involved a clinical examination and blood collection for detection of malaria parasites by microscopy and quantitative polymerase chain reaction. Participants were encouraged to come to the designated study clinic any time that they were feeling unwell. For all routine and unscheduled visits, participants underwent a clinical assessment, and participants with a fever (≥38.0°C) or a history of fever in the past 24 hours had a thick blood smear performed. Those with a positive smear result for malaria parasites were treated with artemether-lumefantrine. At the end of the clinical visit, the clinician recorded any new diagnoses and medications prescribed using standardized diagnostic codes and treatment algorithms.

### Patient Consent Statement

This was a secondary post hoc analysis of deidentified data from a malaria chemoprevention trial in Uganda (NCT02793622 [11]). Written informed consent was obtained from each participant prior to study procedures. Ethical approvals covered the collection of all data used in this article. Ethics approvals for the trial were granted by the Makerere University School of Biomedical Sciences, the Uganda National Council for Science and Technology, and the University of California San Francisco.

### Exposure

Exposure was defined as random assignment to monthly IPTp with SP or DP.

### Outcomes

The outcomes of our study were neonatal birthweight (measured in grams) and “birthweight for gestational age” *Z* score (BWGAZ) based on INTERGROWTH-21st standards for newborn weight [28]. Secondary outcomes were binary measures of the following outcomes: low birthweight defined as birthweight <2500 g and “small for gestational age” defined as birthweight <10th percentile for gestational age based on INTERGROWTH-21st standards.

### Potential Mediators

Mediators of interest were the number of incident NMFI episodes and antibiotic prescriptions. An incident NMFI episode was defined as the presence of a fever (tympanic temperature ≥38.0°C or history of fever within the past 24 hours) with negative thick blood smear result. Episodes preceded by another episode within the past 14 days were not considered incident occurrences. NMFIs were classified by the following groupings of standardized diagnostic codes, corresponding to the human body system primarily affected: respiratory, gastrointestinal, and genitourinary. Their classifications were subsequently verified by cross-referencing the presence of localizing symptoms or abnormal physical examination signs. An incident NMFI episode may contribute to 0, 1, or multiple subcategories depending on how many body systems were affected. The number of antibiotic prescriptions given during the study period were counted for each participant and classified per the third level of the Anatomical Therapeutic Chemical classification system (2023 version) [29]: β-lactams (J01C and J01D), fluoroquinolones (J01M), sulfonamides (J01E), or other antibacterials (J01X). Other prescribed medications were classified as antimalarials (P01B), antiparasitics (P02C), and antivirals (J05A).

### Confounders

A simplified direct acyclic graph was used to represent our causal assumptions among exposure, mediator, and outcomes and to identify potential variables that could confound these relationships (Supplementary Figure 1). As our exposure was randomized, we identified confounders that could bias mediator-outcome relationships. Confounders of interest included maternal age, gestational age at enrollment (in weeks), gravidity, maternal parasitemia at enrollment, fetal sex, education, and household wealth. Gravidity was categorized as primigravidae (first pregnancy) or multigravidae (had at least 1 previous pregnancy). Education was dichotomized as having above or below a primary level of education. Household wealth was determined by principal components analysis of common household items and categorized into tertiles [14].

### Statistical Analyses

First, we compared baseline characteristics between women randomized to IPTp-DP and IPTp-SP using the two-sample *t* test for continuous variables and the χ^2^ test for categorical variables. Linear and log-binomial regression models were used to evaluate exposure-outcome and mediator-outcome associations. Poisson regression with robust standard errors were used if log-binomial models did not converge. Poisson regression was used to evaluate exposure-mediator associations with an offset term to account for person-time between initiation of study drugs and delivery.

Mediation analysis was conducted using a potential outcomes framework [30]. Models were adjusted for confounders based on our directed acyclic graph (Supplementary Figure 1). Analyses were conducted using the *maczic* package in R (version 4.2.1; R for Statistical Computing) [31]. We estimated the following: the indirect effect, defined as the effect of IPTp on birthweight outcomes attributed to our prespecified mediator (ie, the mediated effect); the direct effect, defined as the effect of IPTp on birthweight outcomes not attributed to our prespecified mediator (ie, the nonmediated effect); and the total effect, defined as the sum of the direct and indirect effects (ie, the overall difference in birthweight outcomes between IPTp regimens). We tested for exposure-mediator and exposure-gravidity interactions wherever possible and incorporated interaction terms into our models if the *P* value for interaction of these terms was <.100. We conducted stratified analyses by gravidity, regardless of whether there was evidence of a statistical interaction, and 95% CIs around indirect and direct effects were generated by a quasi-bayesian approach of 1000 simulations.

All *P* values were calculated with two-sided tests, and *P* < .05 was considered statistically significant. All data management and statistical analyses were executed in RStudio with R version 4.2.1.

## RESULTS

**Figure 1.**
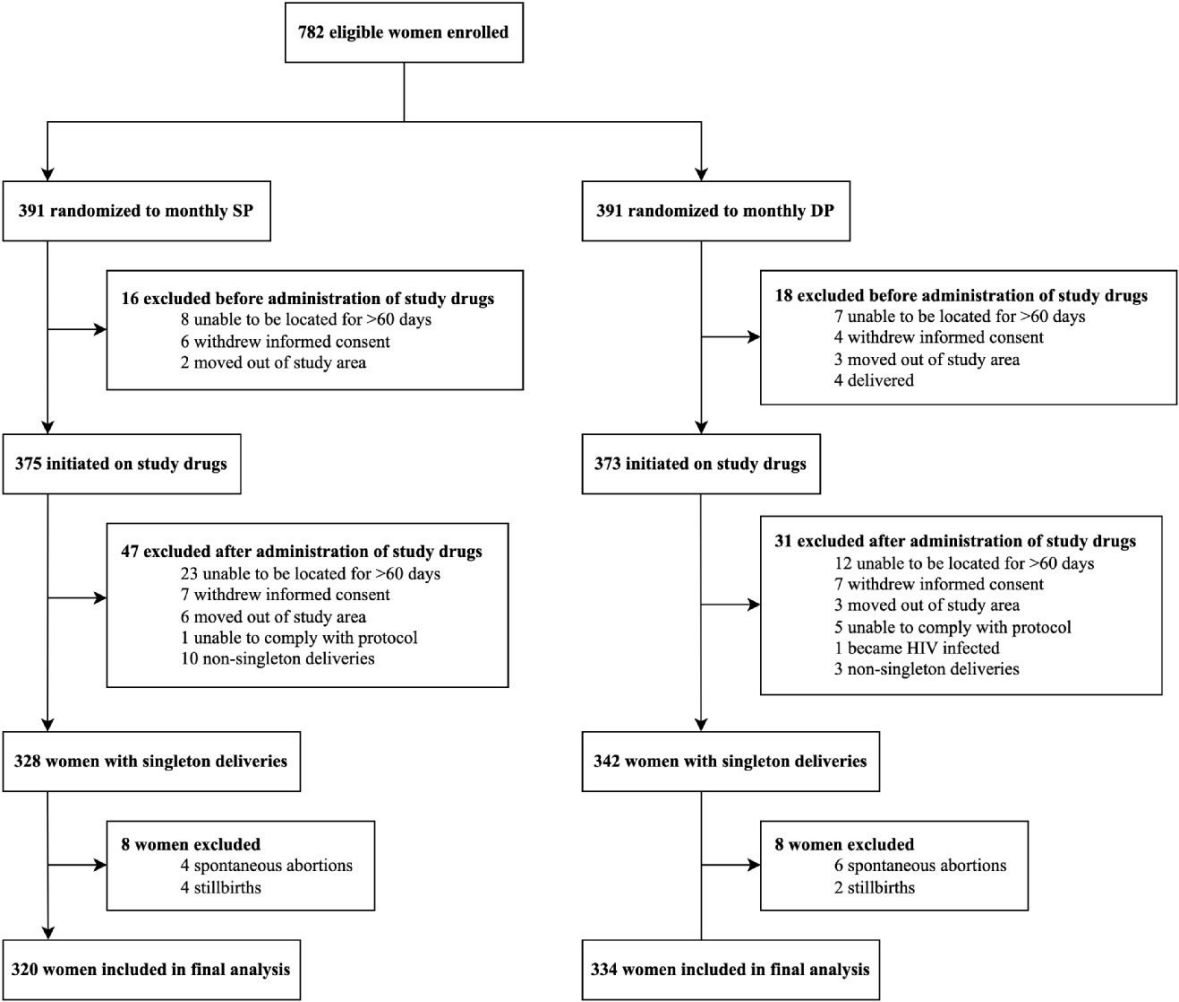
Flowchart of study participants from the Ugandan IPTp trial who were included in our analyses. DP, dihydroartemisinin-piperaquine; IPTp, intermittent preventive treatment in pregnancy; SP, sulfadoxine-pyrimethamine.

Our final analyses included 654 (84%) of the 782 women enrolled in the parent trial [11] (Figure 1). Women were excluded for the following reasons: 34 withdrew before study drugs were given, 65 withdrew before delivery, 13 delivered twins, and 16 experienced fetal loss. After exclusion, 82% (320/391) of the monthly IPTp-SP arm remained and 85% (334/391) of the monthly IPTp-DP arm, with a similar proportion of women who were excluded between arms (*P* = .209). Baseline characteristics were similar between IPTp arms (*P* > .050; Supplementary Table 1). As previously reported [11], adherence to study drugs on days 2 and 3 was >98%, with an average of 6 IPTp courses during the trial.

### Overall Difference in Birthweight Outcomes Between IPTp Regimens

**Figure 2.**
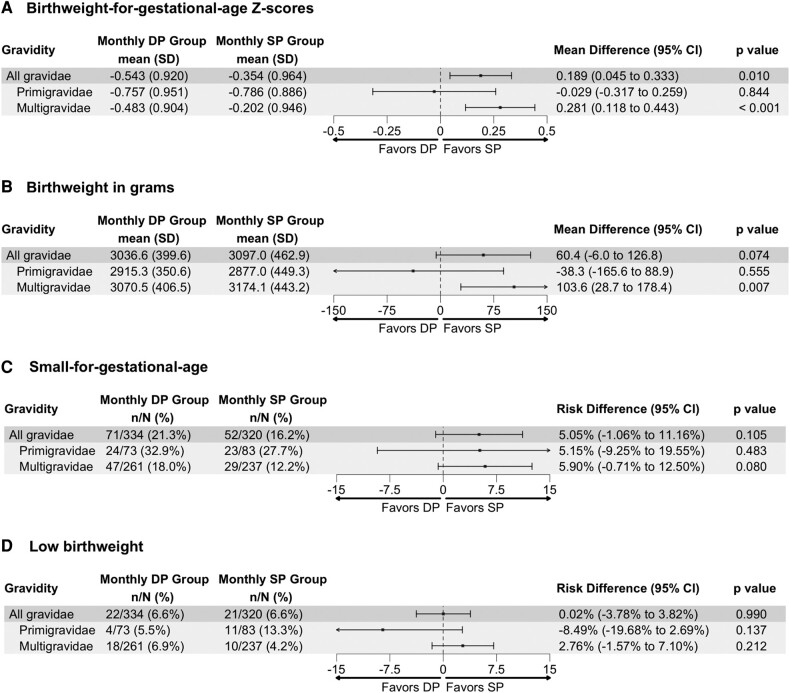
*A–D*, Overall crude difference, stratified by gravidity, between IPTp with SP and IPTp with DP on “birthweight for gestational age” *Z* scores, birthweight, “small for gestational age” risk, and low birthweight risk. DP, dihydroartemisinin-piperaquine; IPTp, intermittent preventive treatment in pregnancy; SP, sulfadoxine-pyrimethamine.

Overall, neonates born to mothers randomized to IPTp-SP had significantly higher BWGAZ as compared with neonates born to mothers randomized to IPTp-DP (mean difference [MD], 0.189; 95% CI, .045–.333; Figure 2*A*). Women randomized to IPTp-SP also had a higher mean birthweight and lower risk of delivering a “small for gestational age” neonate, although these findings did not reach statistical significance (Figure 2*B* and 2*C*). The risk of low birthweight was similar between IPTp arms (risk difference, 0.02%; 95% CI, −3.78% to 3.82%; Figure 2*D*). For all outcomes, the effect estimates favoring IPTp-SP were larger in multigravidae than primigravidae.

### Nonmalarial Febrile Illness

Overall, 337 incident episodes of fever were observed across all participants: 180 in the IPTp-SP arm and 157 in the IPTp-DP arm. The incidence of all-cause febrile illnesses did not significantly differ between groups (incidence rate ratio [IRR], 1.21; 95% CI, .97–1.49). Most fevers were due to respiratory illnesses (34%), malaria (22%), and genitourinary illnesses (21%; Table 1). Women randomized to IPTp-SP experienced a higher incidence of malaria episodes as compared with women in the IPTp-DP arm (IRR, 24.87; 95% CI, 7.84–78.94). In terms of number needed to treat, this equates to needing to administer IPTp-DP instead of IPTp-SP to 2 women to avert 1 malaria episode. In contrast, NMFI incidence was lower in women randomized to IPTp-SP than IPTp-DP (IRR, 0.74; 95% CI, .58–.95), mainly driven by the lower incidence of febrile respiratory illness episodes (IRR, 0.69; 95% CI, .48–1.00; Table 1). In terms of number needed to treat, this translates to 2 women who would need to be administered IPTp-DP instead of IPTp-SP to avert 1 malaria episode, while 4 women would need to be administered IPTp-SP instead of IPTp-DP to avert 1 NMFI of any cause and 7 women to avert 1 respiratory NMFI. No differences in genitourinary febrile illnesses were observed between IPTp arms (IRR, 0.99; 95% CI, .63–1.58; *P* = .980).

**Table 1.** Crude Effects of IPTp Regimens on Febrile Illnesses

	No. of Incident Events (Events per Person-Year at Risk)		SP:DP	
	Monthly SP (n = 320)	Monthly DP^a^ (n = 334)	IRR (95% CI)^b^	*P* Value
Febrile illnesses				
Overall	180 (1.35)	157 (1.11)	1.21 (.97–1.49)	.088
Primigravidae	64 (1.89)	26 (0.88)	2.16 (1.37–3.41)	<.001
Multigravidae	116 (1.17)	131 (1.17)	0.98 (.77–1.26)	.899
Malaria	71 (0.53)	3 (0.02)	24.87 (7.84–78.94)	<.001
Primigravidae	44 (1.30)	1 (0.03)	38.62 (5.32–280.25)	<.001
Multigravidae	27 (0.27)	2 (0.02)	15.00 (3.57–63.08)	<.001
Nonmalarial	109 (0.82)	154 (1.09)	0.74 (.58–.95)	.018
Primigravidae	20 (0.59)	25 (0.84)	0.70 (.39–1.26)	.238
Multigravidae	89 (0.90)	129 (1.15)	0.77 (.59–1.00)	.054
Types of NMFIs				
Respiratory illness	46 (0.35)	70 (0.49)	0.69 (.48–1.00)	.051
Primigravidae	7 (0.21)	9 (0.30)	0.68 (.25–1.83)	.449
Multigravidae	39 (0.39)	61 (0.55)	0.71 (.48–1.06)	.095
Gastrointestinal illness	5 (0.04)	4 (0.03)	1.31 (.35–4.89)	.684
Primigravidae^c^	0 (0.00)	0 (0.00)	…	…
Multigravidae	5 (0.05)	4 (0.04)	1.39 (.37–5.17)	.624
Genitourinary illness	35 (0.26)	37 (0.26)	0.99 (.63–1.58)	.980
Primigravidae	5 (0.15)	9 (0.30)	0.49 (.16–1.45)	.198
Multigravidae	30 (0.30)	28 (0.25)	1.19 (.71–1.99)	.507

Abbreviations: DP, dihydroartemisinin-piperaquine; IPTp, intermittent preventive treatment in pregnancy; IRR, incidence rate ratio; NMFI, nonmalarial febrile illness; SP, sulfadoxine-pyrimethamine.

^a^Reference group.

^b^IRR and 95% CI were calculated by univariate Poisson regression offset by time that each study participant contributed to the final analysis.

^c^IRR and 95% CI cannot be determined because the value of the denominator equals 0 and the upper bound of the 95% CI diverges to infinity.

**Figure 3.**
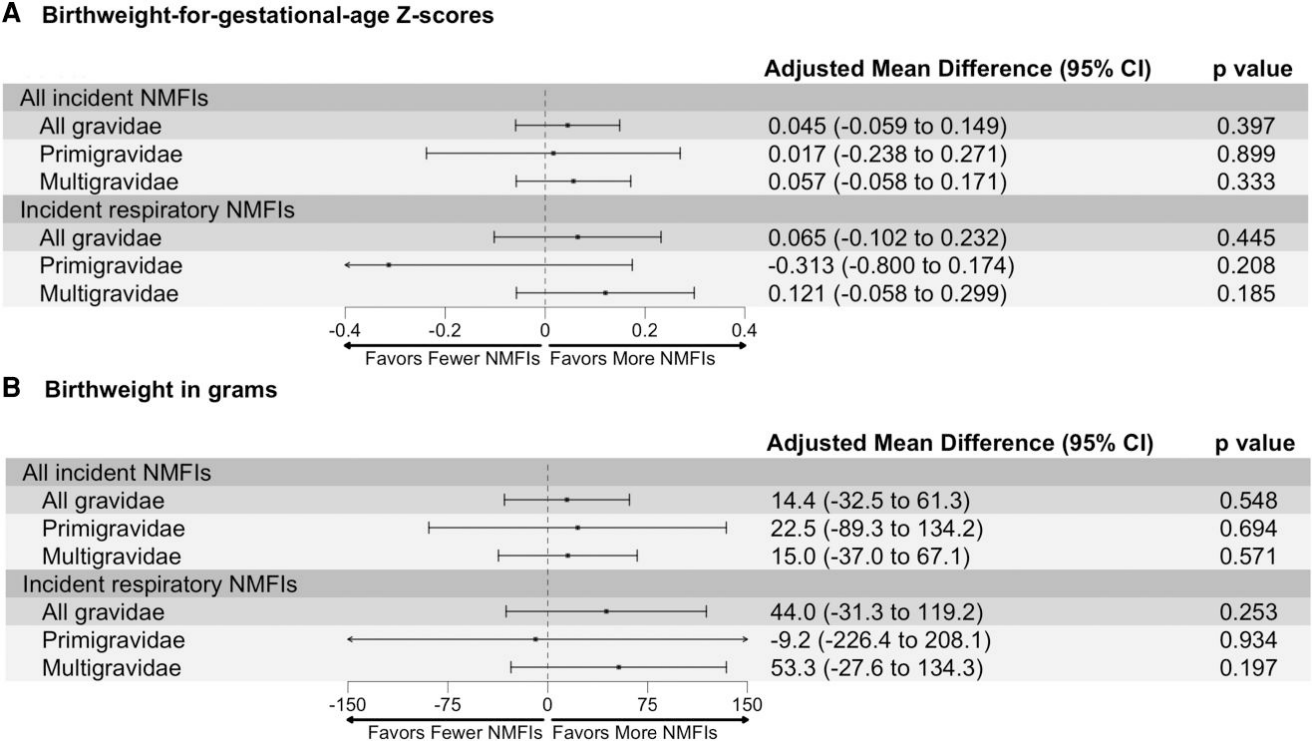
*A* and *B*, Association between all NMFIs and respiratory NMFIs on “birthweight for gestational age” *Z* scores and birthweight, stratified by gravidity. Models were adjusted for the following covariates: maternal age, gestational age at enrollment, gravidity (except for subgroup analyses), maternal parasitemia at enrollment, fetal sex, education, and household wealth. The presented adjusted mean difference is interpreted as the observed difference in outcome for every 1-unit increase in incident NMFIs or incident respiratory NMFIs. NMFI, nonmalarial febrile illness.

Respiratory NMFIs were associated with nonsignificantly lower BWGAZ and birthweight in primigravidae, yet the direction of point estimates suggests a positive relationship in multigravidae: BWGAZ (adjusted MD, primigravidae vs multigravidae: −0.313 [95% CI, −.800 to .174] vs 0.121 [95% CI, −.058 to .299]; interaction *P* = .057) and birthweight (adjusted MD, primigravidae vs multigravidae: −9.2 g [95% CI, −226.4 to 208.1] vs 53.3 g [95% CI, −27.6 to 134.3]; interaction *P* = .060; Figure 3). Thus, while IPTp-SP was associated with a lower incidence of respiratory illness incidence and better birthweight outcomes as compared with IPTp-DP, mediation analyses revealed that prevention of respiratory nonmalarial fevers did not substantively mediate the relationship between IPTp regimens and birthweight outcomes (indirect effect: birthweight, −1.7 g [95% CI, −7.5 to 1.9]; BWGAZ, −0.003 [95% CI, −.015 to .005]; Supplementary Figure 2).

### Antibiotic Prescriptions During Pregnancy

**Figure 4.**
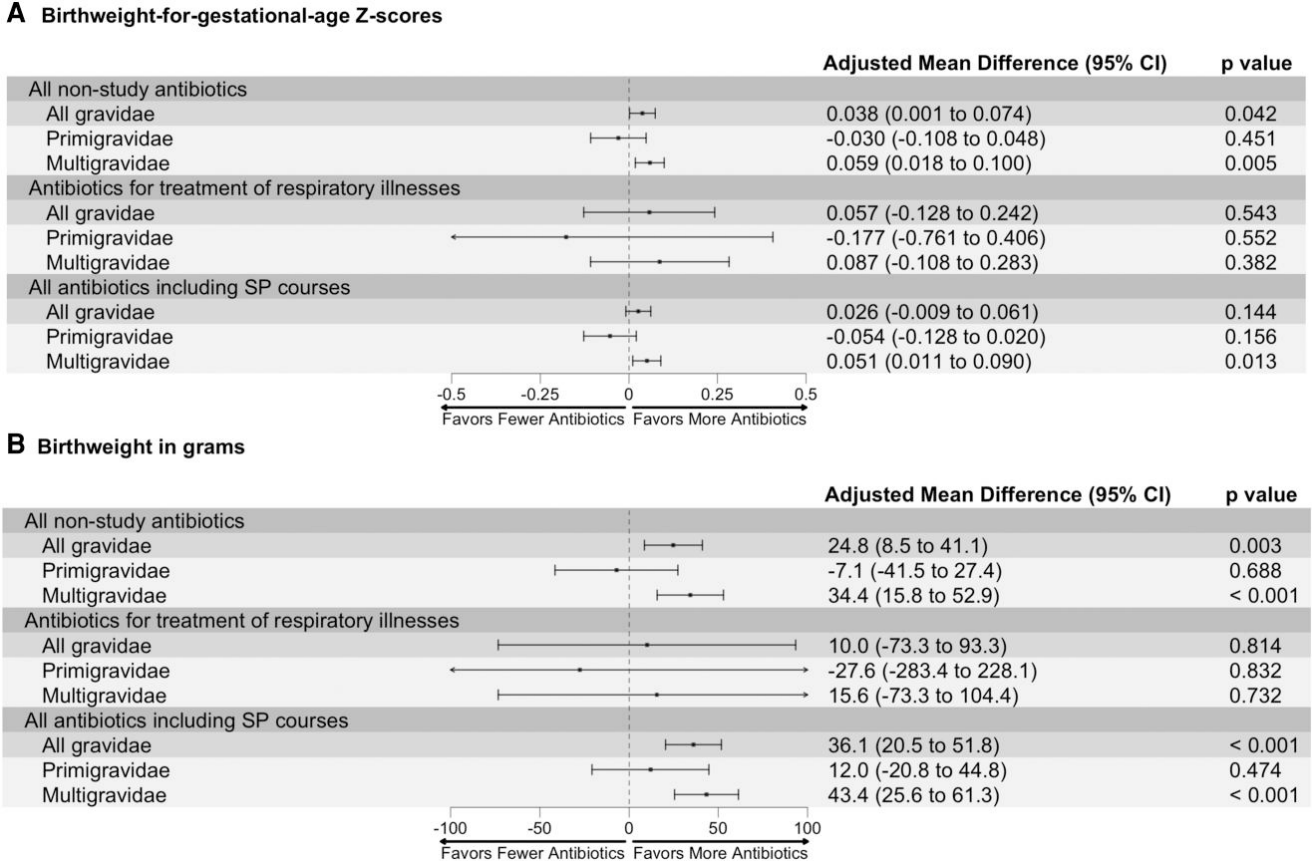
*A* and *B*, Association among nonstudy antibiotic prescriptions, those for treatment of respiratory illnesses, and total antibiotic prescriptions on “birthweight for gestational age” *Z* scores and birthweight, stratified by gravidity. Models were adjusted for the following covariates: maternal age, gestational age at enrollment, gravidity (except for subgroup analyses), maternal parasitemia at enrollment, fetal sex, education, and household wealth. The presented adjusted mean difference is interpreted as the observed difference in outcome for every 1-unit increase in antibiotic prescriptions: those excluding SP courses, those only for the treatment of respiratory illnesses, and those including SP courses. SP, sulfadoxine-pyrimethamine.

We hypothesized that the observed positive relationship between respiratory illnesses and birthweight could be explained by presumptive treatment with antibiotics [27]. In our study, participants were prescribed an average of 2 nonstudy antibiotics (ie, excluding IPTp-SP courses) during the study period (range, 0–12) or about 4 to 5 nonstudy antibiotics per person-year. The incidence of antibiotic prescriptions did not differ between arms (IRR, 0.98; 95% CI, .88–1.09), and no statistically significant interaction with gravidity was observed (interaction *P* = .207; Table 2). Of the 116 incident respiratory illnesses, the majority (75%) were treated with antibiotics, primarily with β-lactams. Adjusted analyses found that the use of nonstudy antibiotics was associated with improved birthweight such that for each antibiotic prescribed, mean BWGAZ increased by 0.038 (95% CI, .001–.074) and mean birthweight increased by 24.8 g (95% CI, 8.5–41.1); positive effects were mainly observed among multigravidae (Figure 4). Mediation analyses to understand the extent to which increased antibiotic exposure mediated the greater impact of IPTp-SP on birthweight outcomes were not conducted given that antibiotic exposure did not differ between arms.

**Table 2.** Crude Effects of IPTp Regimens on Antibiotic Prescriptions

	No. of Prescriptions (Prescriptions per Person-Year at Risk)		SP:DP	
	Monthly SP (n = 320)	Monthly DP^a^ (n = 334)	IRR (95% CI)^b^	*P* Value
Antibacterials				
Overall				
Not including SP courses	616 (4.62)	660 (4.66)	0.98 (.88–1.09)	.732
Including SP courses	2494 (18.71)	660 (4.66)	3.97 (3.65–4.33)	<.001
By antibiotic type				
β-Lactams	572 (4.29)	620 (4.38)	0.97 (.87–1.09)	.595
Fluoroquinolones^c^	0 (0.00)	2 (0.014)	…	.996
Sulfonamides				
Not including SP courses^c^	0 (0.00)	2 (0.014)	…	.996
Including SP courses	1878 (14.09)	2 (0.014)	987 (247–3949)	<.001
Other antibacterials^d^	44 (0.33)	36 (0.25)	1.28 (.83–2.00)	.265
For treatment of respiratory illnesses	33 (0.25)	54 (0.38)	0.64 (.42–0.99)	.045
Antimalarials				
Not including study drug courses	75 (0.56)	7 (0.050)	11.26 (5.19–24.43)	<.001
Including study drug courses	1953 (14.65)	1995 (14.10)	1.03 (.97–1.10)	.37
Antiparasitics	13 (0.098)	14 (0.099)	0.98 (.46–2.08)	.95
Antivirals^c^	2 (0.015)	0 (0.00)	…	.996

Abbreviations: DP, dihydroartemisinin-piperaquine; IPTp, intermittent preventive treatment in pregnancy; IRR, incidence rate ratio; SP, sulfadoxine-pyrimethamine.

^a^Reference group.

^b^IRR and 95% CI were calculated by univariate Poisson regression offset by time that each study participant contributed to the final analysis.

^c^IRR and 95% CI cannot be determined because the value of the denominator equals 0 and/or the upper bound of the 95% CI diverges to infinity.

^d^“Other antibacterials” consisted of metronidazole tablets, injections, and syrup.

## DISCUSSION

Our secondary analysis of a trial in Uganda demonstrated that monthly IPTp-SP was associated with significantly higher BWGAZ when compared with IPTp-DP, despite significantly less malaria burden in the IPTp-DP arm. Furthermore, IPTp-SP was associated with a 26% lower incidence of NMFIs, with majority of the impact stemming from differences in respiratory NMFIs (a 31% reduction). However, counterintuitively, NMFIs were associated with higher birthweights, likely due to the presumptive treatment of NMFIs with nonstudy antibiotics. Thus, mediation analyses could not accurately determine whether prevention of NMFIs mediates IPTp-SP's positive impact on birthweight.

Our research adds to the evidence of SP's nonmalarial benefits [9, 14, 15]. While SP may exhibit these effects through a broad spectrum of antimicrobial activities, including preventing reproductive tract infections [20] such as *C trachomatis* [9], reducing the prevalence of enteroaggregative *E coli* [15], and improving intestinal function [21], our study suggests that prevention of respiratory illnesses may be one of these potential mechanisms. Our data are consistent with an earlier trial of malaria chemoprevention among infants, which demonstrated that while monthly SP was less effective than monthly DP in preventing malaria, SP was more effective than DP at preventing respiratory tract infections [32]. Regarding the potential mechanisms of this protection, SP has a similar mechanism of action to trimethoprim-sulfamethoxazole (cotrimoxazole), which has been shown in vitro to have antibacterial activity against *S aureus* and *S pneumoniae* [16, 33] and which has been used to treat various bacterial infections [18, 19]. Many studies across sub-Saharan Africa found *S pneumoniae* and *Haemophilus influenzae* as 2 highly prevalent causative agents of bacterial respiratory infections [34, 35]. Population-based studies in Taiwan and Israel observed harmful effects of bacterial pneumonia and other respiratory infections on birthweight outcomes [36, 37]. Future analyses confirming the potential pathogens causing respiratory illnesses among pregnant women in malaria-endemic settings and the chemoprevention role of SP on these pathogens are required to validate our findings.

Our findings suggest that antibiotics, particularly β-lactams, and SP confer modest improvements in birthweight and BWGAZ, particularly among multigravidae, who are more likely to have acquired placenta-specific malarial immunity [38] and thus for whom the main causes of febrile illnesses were of nonmalarial etiology. This finding contrasts with a retrospective study conducted among pregnant women in rural Ghana, which found no differences in birthweight between women who did and did not receive antibiotics [39]. However, other antibiotics, such as azithromycin, have been shown to positively affect birth outcomes among pregnant women in lower- to middle-income countries [26, 27, 40]. Even with the potentially positive effect of antibiotics on birthweight, responsible antibiotic prescribing practices should be considered to curb antibiotic resistance, which can rapidly develop with repeated use [41].

Although our study demonstrated that IPTp-SP reduced the incidence of respiratory NMFIs, there may be additional off-target effects of IPTp-SP on subclinical infections or conditions that were not captured in this study. Surprisingly, our study did not find a significant difference in the incidence of genitourinary NMFIs between the IPTp arms despite recent evidence suggesting an increased risk in chlamydia infections among women randomized to the IPTp-DP arm vs the IPTp-SP arm (adjusted risk ratio, 4.17; 95% CI, 2.12–8.19) [9]. However, our study was based on detecting febrile illnesses only, and febrile sexually transmitted infections may not be necessarily required for an effect. In addition, the broad antimicrobial effects of SP may result in the following: alterations to the respiratory, gut [15, 42], and/or vaginal [9] microbiome; reductions in acute and chronic maternal inflammation [43–45]; and increases in maternal or fetal weight gain [9, 15], which may be asymptomatic and otherwise go undetected via passive surveillance. Future studies are needed to explore these potential mechanisms and their relative contributions to downstream maternal and infant outcomes.

This study has limitations. First, due to presumptive antibiotic treatment of NMFIs, we were unable to determine the true relevance of preventing NMFIs. Second, incident febrile episodes may have been underestimated if not all episodes were captured at the study clinic. Women were encouraged to come to the clinic if they felt unwell, and transport and care were free to study participants; thus, we expect that this limitation had a minimal impact on our effect estimates. Third, misclassification of diagnoses of febrile illnesses might have occurred nondifferentially with respect to the randomized treatment arm, likely biasing our effect estimates toward the null. While nonmalarial diagnoses were made by confirmation of microscopy and quantitative polymerase chain reaction during routine visits, those at unscheduled visits underwent only microscopy, potentially overestimating the true number of NMFIs in both treatment arms. Fourth, non-SP antibiotic exposure relied on prescriptions from study clinicians, and compliance was not directly observed. Last, our analyses were limited by a small sample size from a trial conducted in a highly malaria-endemic setting. This may have limited our statistical power to detect true differences of other types of NMFIs between arms, especially ones that were rare. Future analyses pooling data from multiple IPTp studies conducted across varied malaria transmission intensities may help to validate our findings and increase generalizability, but this analysis would require standardization of measuring and reporting outcomes across studies.

In summary, our findings suggest that IPTp-SP results in a greater reduction of respiratory NMFIs as compared with IPTp-DP. While our study was unable to determine whether this reduction was associated with better birth outcomes due to presumptive treatment with antibiotics, our findings provide a foundation for future molecular work to explore the mechanisms underlying this pathway. Moreover, we found that antibiotic exposure for NMFIs was associated with improved birthweight, particularly among multigravidae. Together, our findings support current World Health Organization recommendations for the continued use of IPTp-SP, even in areas with high *P falciparum* resistance to SP. Currently, trials in sub-Saharan Africa assessing the safety and efficacy of other combinations on pregnancy and birth outcomes have been completed or are ongoing (eg, DP + azithromycin, NCT03208179 [9]; DP + metronidazole, NCT04189744; DP + SP, NCT04336189). These studies will further our understanding of the interaction between antimalarials and antibiotics in preventing malaria and nonmalarial illnesses and their combined benefits on improving birth and infant outcomes in malaria-endemic settings.

## Supplementary Material

ofae143_Supplementary_Data

## Supplementary Data

Supplementary materials are available at *Open Forum Infectious Diseases* online. Consisting of data provided by the authors to benefit the reader, the posted materials are not copyedited and are the sole responsibility of the authors, so questions or comments should be addressed to the corresponding author.

## Notes

***Potential conflicts of interest*.** All authors: No reported conflicts.
