# Supplementary material for: Monthly Sulfadoxine-Pyrimethamine During Pregnancy Prevents Febrile Respiratory Illnesses: A Secondary Analysis of a Malaria Chemoprevention Trial in Uganda
Source: Open Forum Infect Dis. 2024 Mar 13;11(4):ofae143. doi: 10.1093/ofid/ofae143 (PMC10995957; doi:10.1093/ofid/ofae143)
Supplement: ofae143_Supplementary_Data [file ofae143_supplementary_data.docx]

**SUPPLEMENTARY APPENDIX**

**Supplementary Figure 1.** Directed acyclic graph.

**
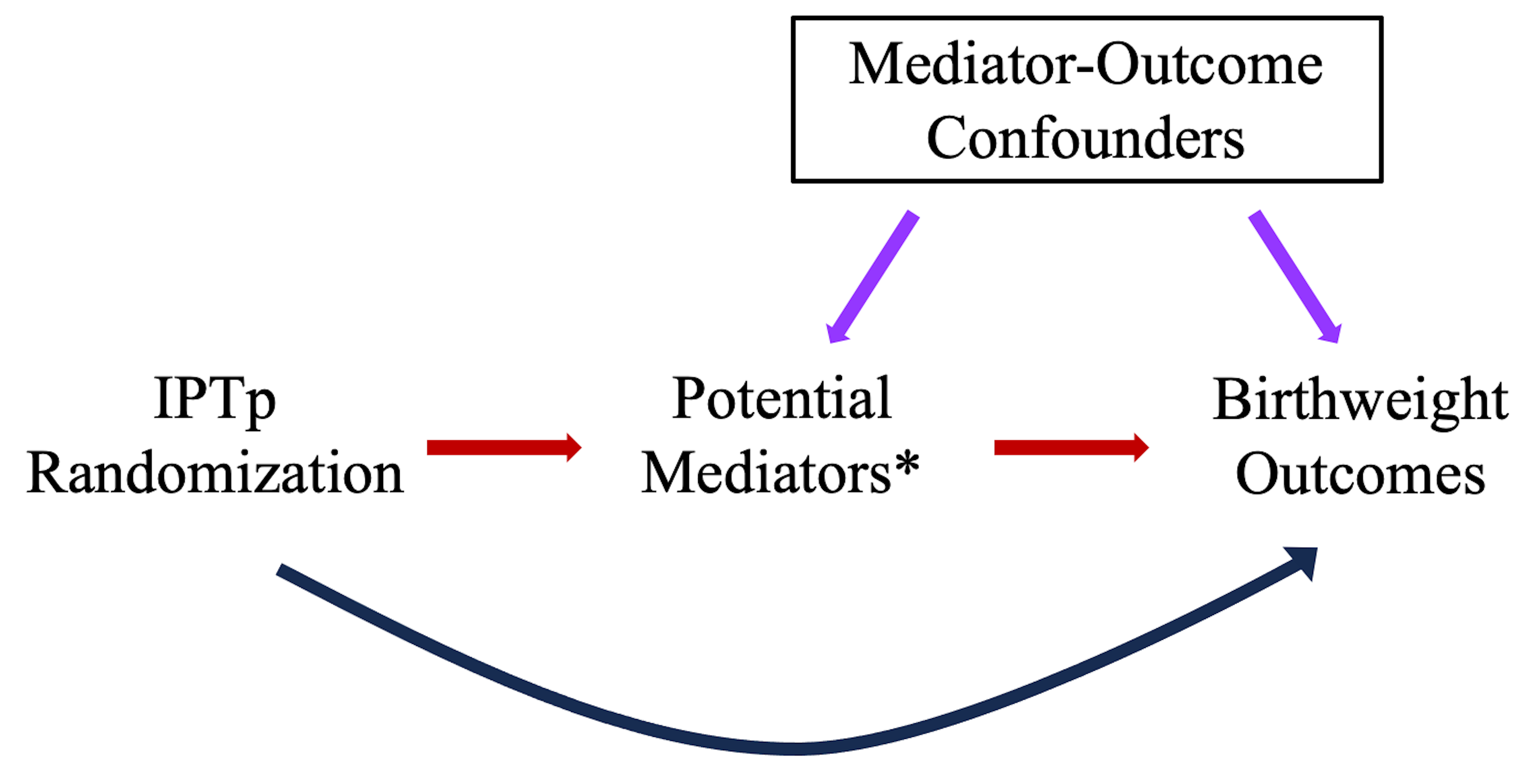
**

Abbreviations: IPTp = intermittent preventive treatment in pregnancy.

*Potential mediators included the number of incident non-malarial febrile illness episodes and antibiotic prescriptions.

**Supplementary Figure 2.** Indirect effect of IPTp regimens, stratified by gravidity, on (A) birthweight-for-gestational-age Z-scores and (B) birthweight, mediated by incident NMFIs and incident respiratory NMFIs. Causal pathway of interest is highlighted in red below.


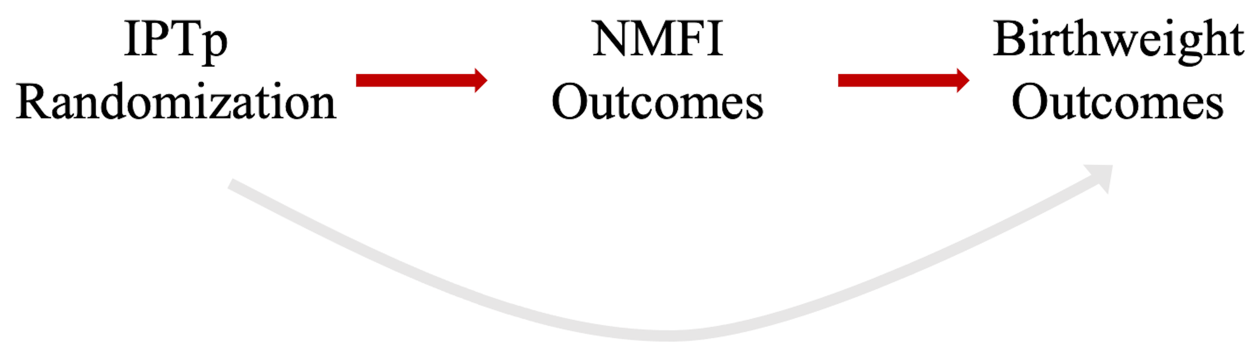


**
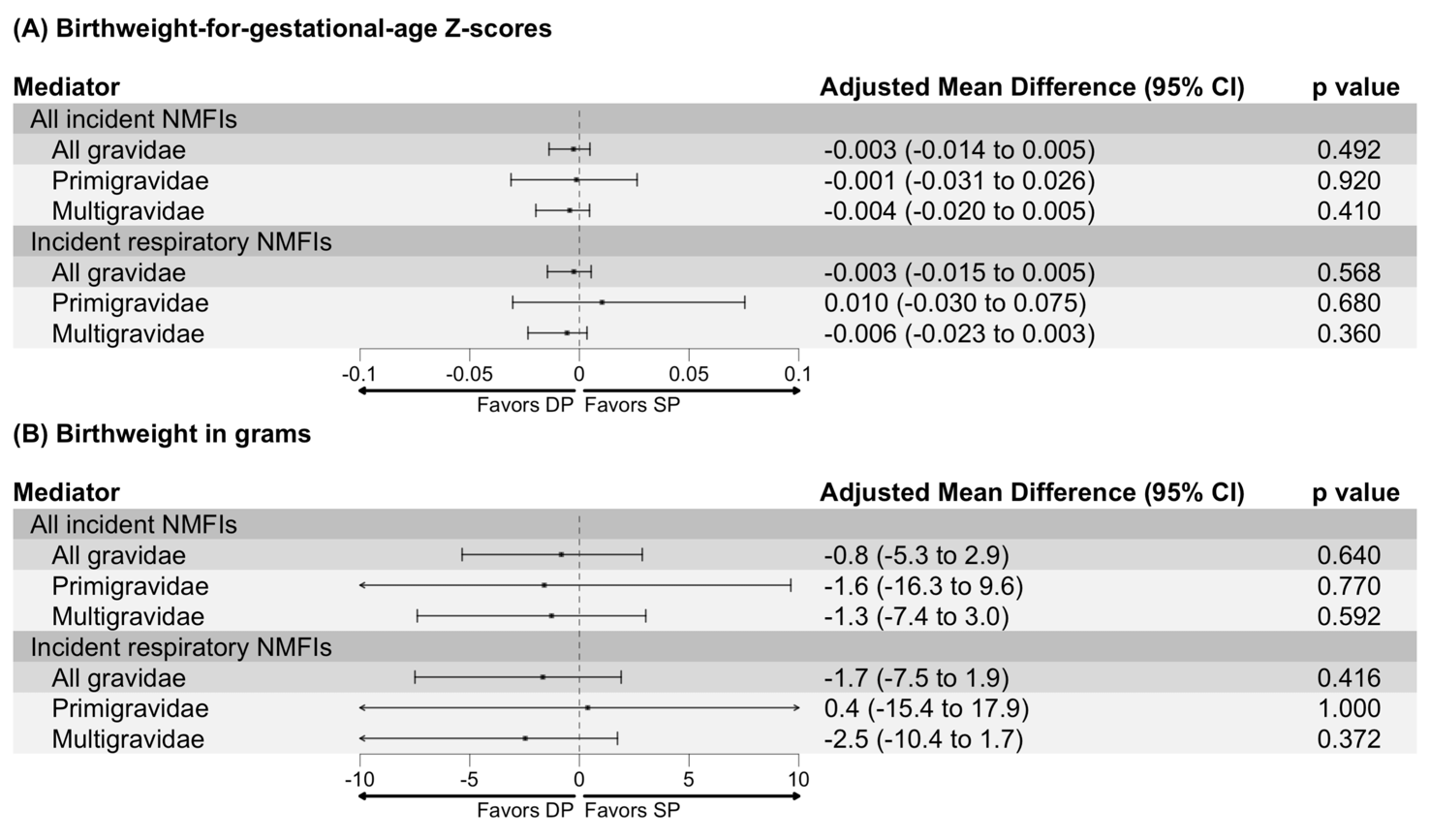
**

Abbreviations: NMFI = non-malarial febrile illness; IPTp = intermittent preventive treatment in pregnancy; CI = confidence interval; DP = dihydroartemisinin-piperaquine; SP = sulfadoxine-pyrimethamine. Models were adjusted for the following covariates: maternal age, gestational age at enrollment, gravidity (except for subgroup analyses), maternal parasitemia at enrollment, fetal sex, education, and household wealth. The presented adjusted mean difference is interpreted as the observed difference in outcome between randomized IPTp arms that is mediated by incident NMFIs or incident respiratory NMFIs.

**Supplementary Table.** Study population characteristics of those included in final analyses (N=654).

|  |  |  |
| --- | --- | --- |
| **Characteristics** | **Monthly SP group**  **(N = 320)** | **Monthly DP group**  **(N = 334)** |
| **Age at enrollment in years, mean (SD)** | 24.4 (6.0) | 24.3 (5.7) |
| **Gestational age at enrollment in weeks, mean (SD)** | 15.7 (2.4) | 15.5 (2.3) |
| **Gestational age category in weeks, n (%)** | | |
| 12 – 16 | 184 (57.5) | 203 (60.8) |
| >16 – 20 | 136 (42.5) | 131 (39.2) |
| **Gravidity category, n (%)** | | |
| Primigravida | 83 (25.9) | 73 (21.9) |
| Multigravida | 237 (74.1) | 261 (78.1) |
| **Wealth category, n (%)** | | |
| Least poor | 109 (34.1) | 104 (31.1) |
| Middle | 103 (32.2) | 113 (33.8) |
| Poorest | 108 (33.8) | 117 (35.0) |
| **Education category, n (%)** | | |
| O-Level or beyond | 74 (23.1) | 81 (24.3) |
| None or primary level | 246 (76.9) | 253 (75.7) |
| **ITN Coverage** | | |
| Ownership of ITN at enrollment, n (%) | 45 (14.1) | 39 (11.7) |
| Reported sleeping under an ITN the previous night before enrollment, n (%) | 36 (11.2) | 37 (11.1) |
| **Detection of malaria parasites by blood smear at enrollment, n (%)** | 161 (50.3) | 179 (53.6) |
| **Detection of malaria parasites by blood smear or qPCR at enrollment, n (%)** | 267 (83.4) | 271 (81.1) |
| **Fetal Sex, n (%)** | | |
| Male | 165 (51.6) | 156 (46.7) |
| Female | 155 (48.4) | 178 (53.3) |
| **Number of Study Drug Courses Given, mean (SD)** | 5.9 (0.8) | 6.0 (0.8) |
|  |  |  |

Abbreviations: SP = sulfadoxine-pyrimethamine; DP = dihydroartemisinin-piperaquine; SD = standard deviation; ITN = insecticide-treated net; qPCR = quantitative PCR.
